# The emerging role of m^6^A methylation in prostate-related diseases: mechanisms and clinical implications

**DOI:** 10.3389/fimmu.2026.1685015

**Published:** 2026-02-12

**Authors:** Qinghua Xie, Hongyan Zhao, Xuan Yang, Hongqi Zhang, Hong Liu, Jinghua Pan, Yan Li, Danping Fan, Xinrong Fan

**Affiliations:** Experimental Research Center, China Academy of Chinese Medical Sciences, Beijing, China

**Keywords:** benign prostatic hyperplasia, m^6^A methylation modification, prostate cancer, prostate-related diseases, prostatitis

## Abstract

Prostate-related diseases, including prostatitis, benign prostatic hyperplasia (BPH), and prostate cancer (PCa), represent significant threats to the health of the aging male population worldwide. Despite their prevalence, the pathogenesis of prostate-related diseases has not been elucidated. Recent studies have shown that N^6^-methyladenosine (m^6^A) modification is widely involved in the progression of prostate-related diseases. In this review, we summarized recent advances in understanding the core m^6^A regulatory machinery comprising writers such as the methyltransferase-like 3 (METTL3)-METTL14 complex, erasers including fat mass and obesity-associated protein (FTO) and AlkB homolog 5 (ALKBH5), and readers, including the YTH domain-containing family proteins (YTHDFs), YTHDC proteins, insulin-like growth factor 2 mRNA-binding proteins (IGF2BPs), and heterogeneous nuclear ribonucleoproteins (HNRNPs). Specifically, we elucidated how dysregulation of these components drives disease progression via alterations in cellular proliferation, differentiation, inflammatory responses, and stem cell dynamics. Notably, m^6^A modifications help shape the immunosuppressive landscape in PCa by modulating immune checkpoint expression, cytokine networks, and immune cell infiltration, thereby critically influencing therapeutic responses to immunotherapy. Furthermore, this review highlights the emerging diagnostic potential and therapeutic viability of m^6^A-targeted strategies, offering valuable insights for future clinical translation in prostate-related diseases.

## Graphical Abstract

**Figure:**
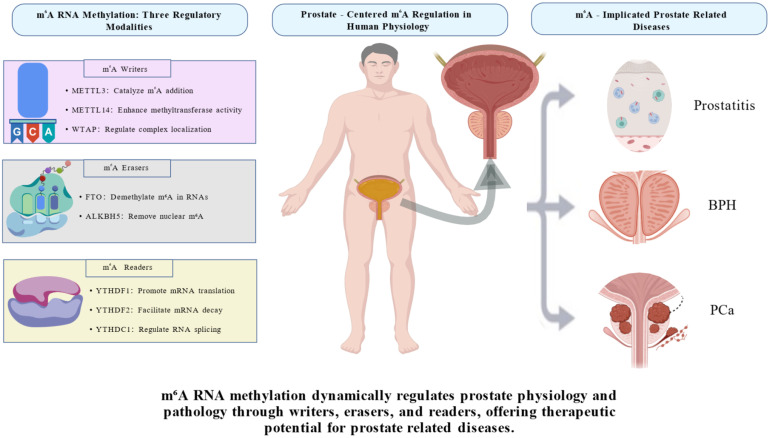
N^6^-methyladenosine (m^6^A) RNA methylation dynamically orchestrates prostate physiology and pathology through the coordinated actions of writers (e.g., METTL3, METTL14, and WTAP), erasers (FTO and ALKBH5), and readers (YTHDF1, YTHDF2, YTHDC1), which together regulate mRNA stability, splicing, translation, and decay. This epitranscriptomic machinery modulates key cellular processes, including proliferation, differentiation, inflammatory responses, and stemness, within the prostate gland, as illustrated by the central anatomical depiction of the human prostate. Dysregulation of m^6^A modification drives a pathological continuum: in prostatitis, aberrant m^6^A signaling exacerbates chronic inflammation via altered cytokine production and immune cell infiltration; in benign prostatic hyperplasia (BPH), it promotes stromal and epithelial hyperplasia by enhancing cell growth and suppressing apoptosis; and in prostate cancer (PCa), m^6^A imbalance fosters tumor progression through oncogene activation, tumor suppressor silencing, and the establishment of an immunosuppressive microenvironment, characterized by upregulated immune checkpoints (e.g., PD-L1) and impaired T-cell infiltration. Collectively, this visual narrative positions m^6^A methylation as a pivotal epigenetic switch across the spectrum of prostate-related diseases, highlighting its emerging potential as a source of diagnostic biomarkers and therapeutic targets in precision oncology.

## 1 Introduction

Prostate-related diseases have been established as the main threats to the health of elderly men worldwide, with prostatitis, benign prostatic hyperplasia (BPH), and prostate cancer (PCa) being the three most common types of diseases (1). Prostatitis is one of the most common diseases diagnosed in urological clinics worldwide, especially prevalent in men aged 20–50 years, with an estimated prevalence rate of up to 5%-10%. This inflammatory state, characterized by pelvic pain and abnormal urination, not only seriously affects patient quality of life but is also believed to be closely related to the subsequent development of BPH and PCa, although its specific mechanism remains poorly understood (2). BPH is the most common cause of urinary diseases in middle-aged and elderly men, and its incidence increases exponentially with age. More than 50% of men will experience related pathological changes at the age of 60, and up to 83% at the age of 80 (3). Worldwide, lower urinary tract symptoms caused by BPH are among the most common complaints in urology (4). PCa is the second most common malignant tumor among men worldwide and the fifth leading cause of cancer-related deaths (5). Epidemiological data suggest that the incidence of PCa is highest in North America, Europe, and Australia, but it is also increasing rapidly in developing countries (6). As the population ages and life expectancy increases, the World Health Organization predicts that the global burden of PCa will continue to increase over the following decade (7).

The pathogenesis of prostate-related diseases has traditionally focused on genetic factors. In recent years, a large number of studies have shown that epigenetic regulation, especially the abnormal modification of N^6^-methyladenosine (m^6^A) and the dysfunction of its regulatory factors including methyltransferase complex (MTC), demethylase, and m^6^A binding protein, is widely involved in the expression regulation of prostate specific genes, such as androgen receptors (AR), inflammation related factors, and cell proliferation regulatory genes, playing a key role in the occurrence and development of prostatitis, BPH, and PCa (8–10). Crucially, m^6^A-mediated metabolic reprogramming sustains tumor proliferation and tissue adaptation, which is particularly relevant to PCa progression, where altered lipid metabolism fuels aggressive phenotypes (11). Concurrently, m^6^A dysregulation modulates drug-metabolizing enzymes and efflux transporters, directly impacting PCa treatment resistance to AR inhibitors or taxanes (12). Furthermore, m^6^A governs adaptive responses such as apoptosis evasion and immune escape pathways (12), mechanisms implicated in prostatitis chronicity and BPH inflammatory persistence. Growing evidence suggests that m^6^A modification and its regulatory factor network may provide new molecular targets and biomarkers for the diagnosis and treatment of prostatitis, BPH, and PCa (13).

This review systematically summarizes the key roles and molecular mechanisms of m^6^A modification in the pathogenesis of prostatitis, BPH, and PCa. Emphasis is placed on the specific expression patterns and functional characteristics of m^6^A regulatory factors across different prostate-related diseases, as well as the potential value of m^6^A modification for disease diagnosis, prognosis evaluation, and treatment intervention. By exploring the complex regulatory network of m^6^A modification in prostate-related diseases, we aim to provide a theoretical foundation and practical guidance for future translational medicine research.

## 2 Pathological mechanism links among prostatitis, BPH, and PCa

Prostate-related diseases, encompassing prostatitis, BPH and PCa, constitute a continuous spectrum of pathophysiological processes that exert a profound impact on men’s health across diverse age cohorts. These three diseases represent the most prevalent afflictions of the prostate gland, frequently sharing common risk factors (e.g., aging, chronic inflammation) and underlying molecular mechanisms, with significant epidemiological and mechanistic interconnections that establish a complex prostatitis-BPH-PCa axis (2).

Prostatitis, predominantly affecting men aged 20–50 years, often serves as an inciting factor. The chronic inflammatory milieu generated by prostatitis fosters a microenvironment characterized by sustained activation of critical signaling pathways, particularly NF-κB. This activation drives the release of potent pro-inflammatory cytokines such as interleukin-6 (IL-6), IL-8, tumor necrosis factor (TNF)-α, and transforming growth factor (TGF) -β. This cascade induces substantial oxidative stress, DNA damage, and disruption of normal cellular homeostasis within the prostate microenvironment. Crucially, this chronic inflammatory state provides fertile pathological ground not only for the initiation of BPH but also for priming the prostate for PCa development by fostering genomic instability and creating a pro-tumorigenic microenvironment (2).

BPH, which affects more than 50% of men aged 60 and 83% of those aged 80 years (3), is far more than a benign enlargement driven by aging and hormonal influences. Chronic inflammation, often stemming from or exacerbated by prior prostatitis, is a key driver of BPH pathogenesis. Beyond glandular enlargement, long-standing BPH triggers profound tissue remodeling processes. These include epithelial-mesenchymal transition (EMT) and dysregulation of the AR signaling axis, both fueled by the ongoing inflammatory environment. A critical emerging mechanism linking BPH to potential malignancy involves dysregulation of m^6^A RNA methylation. Studies have demonstrated that abnormal m^6^A dynamics can contribute to localized pathological progression within BPH. For instance, specific m^6^A signatures are correlated with the development of pre-cancerous lesions such as proliferative inflammatory atrophy (PIA) and high-grade prostatic intraepithelial neoplasia (HGPIN). Notably, m^6^A-mediated suppression of the tumor suppressor PTEN expression has been implicated in this progression towards a neoplastic phenotype within the context of BPH (9). Furthermore, specific regulators, including the m^6^A demethylase AlkB homolog 5 (ALKBH5), have been directly implicated in modulating the progression from BPH towards more advanced stages (13).

PCa development often follows a discernible sequence within the prostatitis-BPH-PCa continuum, manifesting as inflammation-hyperplasia-carcinogenesis. Chronic inflammation, a common denominator originating from prostatitis and persisting in BPH, plays a pivotal dual role in the pathogenesis of PCa. First, it fuels carcinogenesis through pathways that include inflammation-induced activation of potent oncogenes such as MYC and continued aberrant activation of AR, facilitating malignant transformation (13). Second, inflammation remodels the epigenetic landscape, particularly impacting m^6^A modification, which subsequently modulates key oncogenic drivers. A prime example is the m^6^A-dependent overexpression of versican (VCAN), promoted by the inflammatory environment, which critically enhances tumor cell invasion and metastasis (14). Besides, metabolic disorders frequently associated with BPH can synergistically accelerate PCa carcinogenesis. This acceleration is mechanistically linked to dysregulations in m^6^A dynamics, such as those modulated by enzymes such as ALKBH5 (13). Thus, inflammation serves as a persistent catalyst, promoting hyperplasia through chronic epithelial and stromal stimulation, creating genomic instability, and driving molecular alterations (including m^6^A dysregulation) that collectively facilitate the transition to invasive cancer.

In recent years, epigenetic modifications, particularly m^6^A methylation, have attracted significant interest for their role in the progression of prostate-related diseases. As the most prevalent RNA modification, m^6^A regulates key signaling pathways involved in inflammation, proliferation, and tumorigenesis by modulating mRNA stability, splicing, and translation efficiency. During the transition from BPH to PCa, dysregulated expression of the m^6^A demethylase ALKBH5 may suppress the generation of AR-v7 splice variants by stabilizing SIAH1 mRNA or maintain the stability of the tumor suppressor gene CLIC4 mRNA through an m^6^A-dependent mechanism, thereby modulating tumor progression (15, 16). Furthermore, the m^6^A reader protein YTH domain family 2 (YTHDF2) facilitates tumor metastasis by recognizing and degrading m^6^A-modified tumor suppressor mRNAs, subsequently activating the Akt signaling pathway (17). These findings underscore the central role of m^6^A methylation in the network of prostate-related diseases, highlighting the need for further exploration of its molecular interaction mechanisms and clinical translational potential.

**Figure 1:**
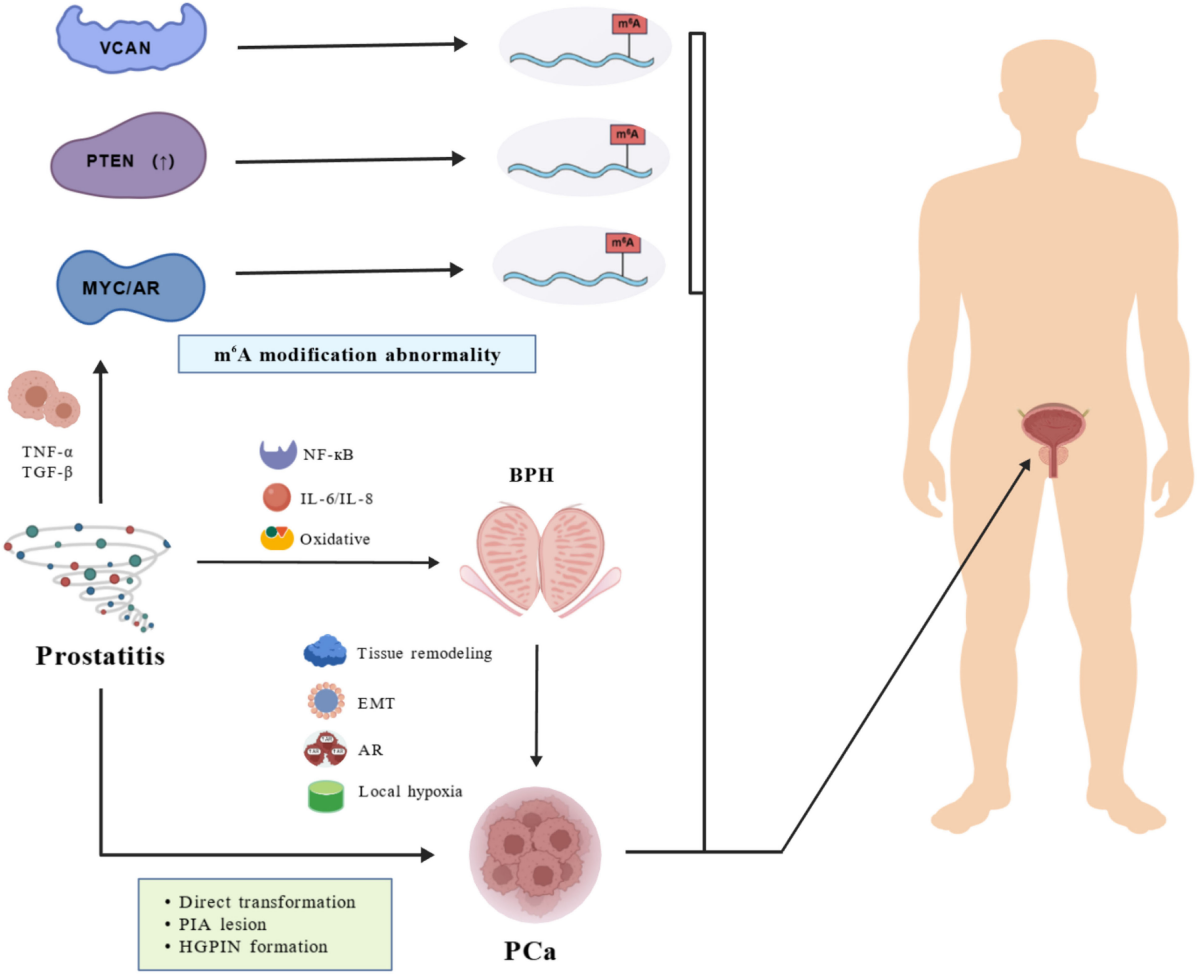
The m^6^A-mediated pathological continuum from prostatitis to BPH and PCa. This schematic illustrates the progressive pathophysiological transition from prostatitis to BPH and ultimately to PCa, driven by chronic inflammation and dysregulated m^6^A RNA methylation. Prostatitis, characterized by persistent inflammatory infiltration, activates key signaling pathways including NF-κB, IL-6/IL-8, and oxidative stress, leading to the release of pro-inflammatory cytokines such as TNF-α and TGF-β. This inflammatory milieu not only disrupts cellular homeostasis but also induces abnormal m^6^A modification patterns in critical regulatory molecules, specifically, upregulation of versican (VCAN), downregulation of the tumor suppressor PTEN, and aberrant activation of MYC and androgen receptor (AR), which collectively promote epithelial damage and tissue remodeling. These molecular alterations serve as a foundation for the development of BPH, marked by glandular enlargement, stromal proliferation, and epithelial-mesenchymal transition (EMT). In BPH, local hypoxia and sustained AR signaling further drive pathological progression, facilitating the emergence of pre-neoplastic lesions such as proliferative inflammatory atrophy (PIA) and high-grade prostatic intraepithelial neoplasia (HGPIN). Crucially, m^6^A dysregulation—particularly through demethylases like ALKBH5—plays a pivotal role in this transition by modulating oncogenic pathways and suppressing tumor-suppressive functions. Ultimately, the cumulative effects of chronic inflammation, genetic instability, and epigenetic reprogramming culminate in malignant transformation into PCa. The figure highlights how m^6^A methylation acts as a dynamic epigenetic regulator across the prostatitis–BPH–PCa axis, mediating crosstalk between inflammatory, hyperplastic, and malignant stages by influencing gene expression, cell fate decisions, and tumor microenvironment remodeling.

The intricate interplay among prostatitis, BPH, and PCa involves multi-layered crosstalk, in which chronic inflammation acts as a central driver, progressively shaping the molecular and cellular landscape toward malignancy through interconnected pathways that encompass oxidative stress, cytokine signaling, altered androgen response, EMT, and specific dysregulation of RNA modifications, such as m^6^A. A conceptual framework illustrating this prostatitis-BPH-PCa continuum, highlighting the central role of chronic inflammation and the progressive dysregulation of key molecular pathways (including m^6^A methylation) that drive disease evolution, is presented in **Figure 1**.

## 3 Overview of m^6^A methylation modification

### 3.1 The concept of m^6^A methylation modification

m^6^A methylation refers to the methylation of the N^6^ position of adenosine residues within RNA molecules. Similar to the mechanism of DNA methylation, RNA methylation is catalyzed by methyltransferases that transfer methyl groups from S-adenosylmethionine (SAM), the primary methyl group donor, to specific methylation sites on RNA (18). In most organisms, messenger RNA (mRNA) is methylated at the 5’ cap end, and its functions include polyadenylation, regulation of mRNA precursor splicing, trafficking and translation initiation, and maintenance of mRNA structure stability. The methylation modification at the polyadenylation tail of the RNA3’ end helps mRNA nuclear transport, translation initiation, and together with polyadenylation binding proteins maintains the stability of mRNA structure (19).

### 3.2 Molecular regulation mechanism of m^6^A methylation modification

The m^6^A methylation modification process is dynamic and reversible, and its biological effects are mainly regulated by MTC, demethylases, and methylation readers (20).

#### 3.2.1 MTC

MTC is a protein complex involved in catalyzing adenosine m^6^A modification of target RNA. Its role is to write methylation modifications onto specific RNA molecules, thereby mediating RNA methylation. MTC proteins mainly include methyltransferase-like protein (METTL14), METTL3, zinc finger CCCH type 13 protein (ZC3H13), vir-like m^6^A methyltransferase-associated protein (VIRMA), Wilms tumor 1-associated protein (WTAP), and RNA binding motif protein 15 (RBM15). These proteins play important roles in MTC to ensure correct m^6^A methylation on target RNA molecules, thereby regulating RNA stability and structural function (21, 22).

Notably, the MTC core member METTL3, the key executor of the “methylation switch”, exhibits abnormally high expression in tumors (e.g., glioma), where it directly activates pro-oncogenic signaling pathways by stabilizing oncogene mRNA transcripts such as EGFR (21). This suggests MTC may drive disease progression through similar mechanisms in prostate-related diseases. Other studies have demonstrated that regulatory factors, including METTL16, RBM15B, and E3 ubiquitin ligase adaptors, are also involved in m^6^A methylation (22, 23). During m^6^A methylation modification, WTAP serves as the methylation scaffold, and SAM acts as the methyl donor, while MTC catalyzes RNA methylation. The core of MTC is a functional heterodimer of METTL3 and METTL14. METTL14 ensures substrate specificity by recognizing target RNA structural motifs (21), while METTL3 catalyzes methyl-group transfer from SAM to adenosine. This synergy regulates translation efficiency physiologically and enables pathological “methylation switches”. METTL14 also maintains METTL3’s structural stability and bioactivity (24). Besides, complementary factors (VIRMA, RBM15) cooperate to complete m^6^A modification (19). Their dysregulation can reshape the tumor microenvironment in PCa. Specifically, VIRMA sustains global m^6^A levels to stabilize oncogenic long non-coding RNA (lncRNA), thereby promoting tumor progression (25).

#### 3.2.2 Demethylases

The role of demethylases is to eliminate RNA methylation signals by directly demethylating RNA. Currently, known m^6^A demethylases include ALKBH5 and fat mass and obesity-associated protein (FTO). FTO is a member of the Fe^2+^/α-ketoglutarate (α-KG)-dependent dioxygenase AlkB subfamily. It requires Fe^2+^ and α-KG as cosubstrates, oxidizing the N-methyl group at m^6^A sites to a hydroxymethyl group, thereby facilitating the removal of the methyl group (26).

#### 3.2.3 Methylation reader

Methylation readers are a class of protein molecules that can specifically recognize m^6^A modification on RNA. They play a key role in the occurrence and development of prostate-related diseases by binding m^6^A sites to regulate the stability, translation efficiency, splicing, and localization of RNA (27). According to the recognition mechanism and functional differences, methylation readers are primarily divided into the following three categories.

##### 3.2.3.1 YTH domain family and nuclear-localized readers

YTH domain proteins represent the first identified and most comprehensively studied m^6^A readers. Interestingly, these proteins can directly recognize and bind m^6^A-modification sites through the C-terminal YTH domain, and then recruit downstream effectors to regulate RNA fate.

Among them, YTH domain family 1 (YTHDF1) is an essential m^6^A recognition protein, which can directly enhance the translation efficiency of target mRNA by binding to the m^6^A site of the 3′ untranslated region (3′ UTR) of mRNA and recruiting the eukaryotic initiation factor 3 (eIF3) complex (28). This mechanism is particularly important in the nervous system because m^6^A modification plays a key role in regulating neuronal function and mRNA metabolism. YTHDF1 not only plays a role in the brain, but also maintains visual function by regulating the translation of TULP1 and DHX38 in the retina (29). In addition, YTHDF1 and other YTHDF family members, such as YTHDF2 and YTH domain family 3 (YTHDF3), participate in the metabolic process of m^6^A-modified mRNA. YTHDF2 mainly accelerates the degradation of m^6^A-modified transcripts, while YTHDF3 cooperates with YTHDF1 to promote protein synthesis and affect YTHDF2-mediated degradation of methylated mRNA (30). In gastric cancer cells, YTHDF2 inhibits cell growth by regulating the FOXC2 signaling pathway, showing its potential therapeutic value in cancer (31). YTHDF3 also plays an essential role in anticancer drug resistance. For example, in oxaliplatin-resistant colorectal cancer cells, YTHDF3 promotes the recruitment of eIF3 by recognizing significantly m^6^A-methylated RNA, thereby regulating the translation of these target genes (32). This mechanism reveals the molecular function of YTHDF3 in tumor drug resistance and provides a new target for anticancer therapy.

Furthermore, nuclear-localized YTH domain proteins, including YTHDC1 and YTHDC2, play critical roles in m^6^A-mediated gene regulation. YTHDC1, a protein localized to the nucleus, can recognize and bind m^6^A-modified RNA molecules. In PCa, YTHDC1 regulates AR transcriptional activity by interacting with AR, thereby affecting PCa progression and treatment resistance. It also cooperates with epigenetic regulators such as KDM4C to promote chromatin modification and gene expression regulation (33). YTHDC2, a protein with RNA helicase activity, can regulate RNA structure and stability. In PCa metastasis, YTHDC2 expression is abnormally elevated, which may enhance the invasion and metastasis ability of cancer cells (34). It affects the proliferation and migration of cancer cells by regulating m^6^A modification of specific genes and may participate in cancer progression and treatment resistance by modulating cellular signaling pathways such as the NF-κB pathway (34). Beyond PCa, YTHDC1 has been implicated in hepatocellular carcinoma, where it promotes cancer cell growth and metabolic reprogramming by regulating lipid metabolism-related genes (35), while YTHDC2 affects the pathophysiology of epilepsy by modulating glutamate transporter expression (36).

**Figure 2:**
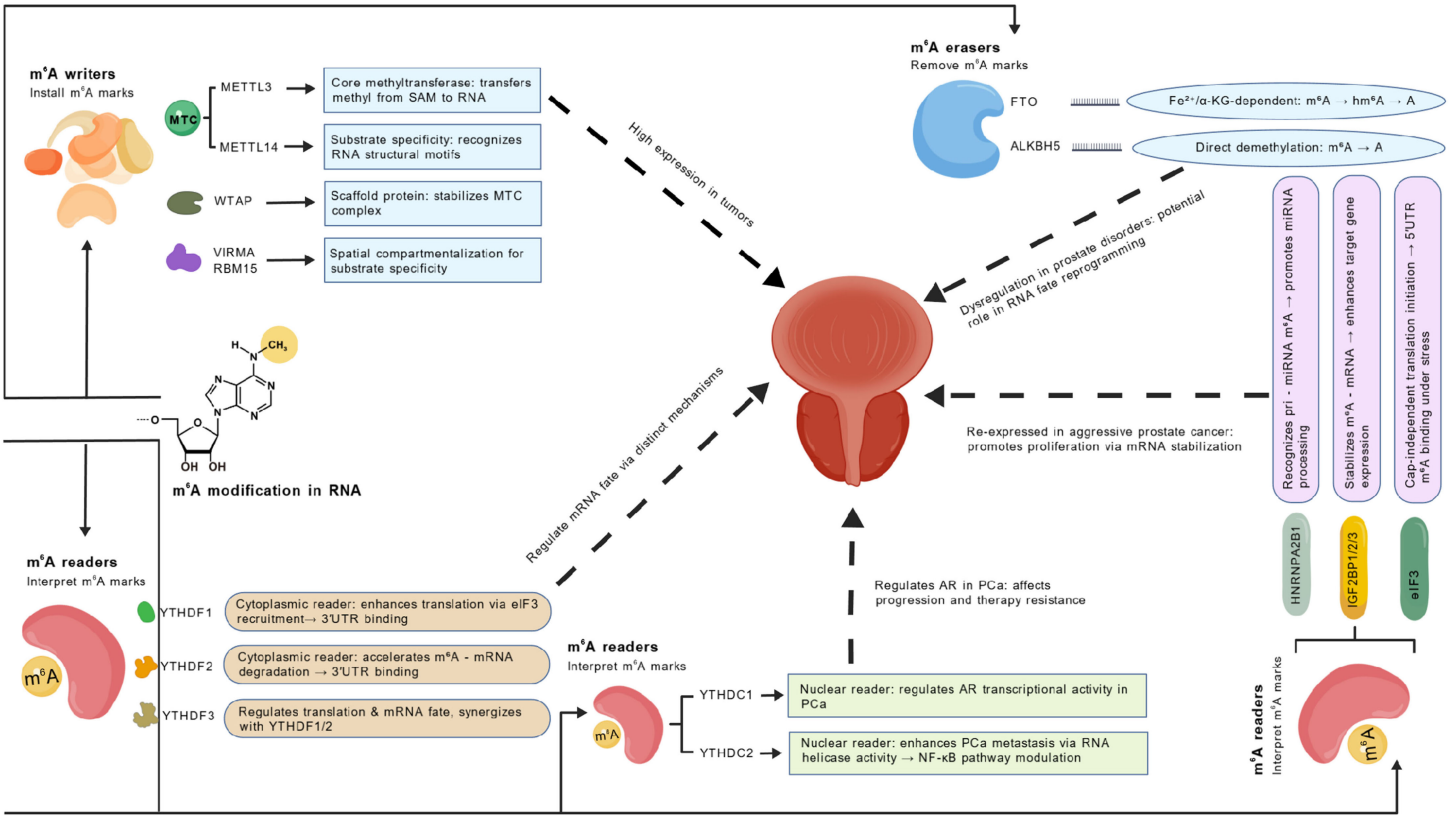
Molecular mechanisms of m^6^A methylation and its functional roles in prostate-related diseases. This schematic depicts the dynamic regulation of m^6^A RNA methylation by writers, erasers, and readers, and their implications in prostatitis, BPH, and PCa. The methyltransferase complex (MTC), comprising METTL3 (catalytic subunit), METTL14 (substrate recognizer), and WTAP (scaffold), adds m^6^A marks using S-adenosylmethionine (SAM) as the methyl donor; auxiliary factors VIRMA and RBM15 confer spatial specificity. Elevated METTL3 promotes PCa by stabilizing oncogenic transcripts. Demethylation is mediated by FTO (an Fe^2+^/α-KG-dependent dioxygenase) and ALKBH5; loss of ALKBH5 exacerbates disease progression. m^6^A marks are interpreted by reader proteins: cytoplasmic YTHDF1 enhances translation, YTHDF2 promotes mRNA decay, and YTHDF3 modulates both processes. Nuclear readers YTHDC1 and YTHDC2 regulate splicing, chromatin remodeling, and AR signaling—YTHDC1 directly interacts with AR to drive PCa progression, while YTHDC2 enhances metastasis via NF-κB activation. Dysregulation of this m^6^A machinery alters RNA fate, reprograms AR signaling, and contributes to inflammation, hyperplasia, and malignant transformation across the prostate-related diseases spectrum.

In summary, YTH domain proteins and nuclear-localized readers collectively regulate m^6^A-mediated RNA fate through diverse mechanisms. YTHDF family proteins primarily modulate mRNA translation and degradation in the cytoplasm, while YTHDC1 and YTHDC2 govern transcriptional regulation, chromatin remodeling, and RNA structural dynamics in the nucleus. These findings not only deepen our understanding of m^6^A modification in gene expression regulation but also provide new perspectives for diagnosing and treating cancers (**Figure 2**).

##### 3.2.3.2 Non-YTH family readers

It is now understood that m^6^A readers with non-YTH domains recognize RNA modifications through unique binding mechanisms and regulate RNA metabolic processes independently of canonical YTH domains. These readers encompass diverse protein families that fine-tune RNA fate through distinct pathways.

Among them, the heterogeneous nuclear ribonucleoprotein (HNRNP) family is well-characterized and includes members such as HNRNPA2B1, HNRNPC, and HNRNPG. This family plays a pivotal role in RNA metabolism, where HNRNPA2B1 recognizes m^6^A modifications on primary miRNAs (pri-miRNAs) and recruits the DROSHA-DGCR8 complex to promote miRNA maturation (37). Other HNRNP members, including HNRNPC and HNRNPG, participate in mRNA splicing and subcellular localization, thereby expanding the regulatory scope of m^6^A recognition. Beyond the HNRNP family, the insulin-like growth factor 2 mRNA-binding protein (IGF2BP) family serves as another critical class of m^6^A readers. IGF2BPs are highly conserved RNA-binding proteins (38) that stabilize m^6^A-modified mRNAs and enhance target gene expression (39). For instance, IGF2BP1 and IGF2BP3 are re-expressed in aggressive cancers, where they regulate cellular proliferation and differentiation by binding target mRNAs, confirming their role in transcript stabilization (40). In addition, the eIF3 complex contributes to translation initiation by binding mRNA and facilitating ribosomal subunit assembly (41, 42). Moreover, eIF3 can initiate cap-independent translation via interactions with m^6^A sites in the 5’UTR, a mechanism particularly important under cellular stress. Another reader, fragile X messenger ribonucleoprotein (FMRP), functions as a multifunctional protein in neuronal development and synaptic plasticity. Interestingly, FMRP interacts with m^6^A-modified mRNAs to modulate their translation, thereby influencing neuronal function (43, 44).

Finally, proline-rich coiled-coil 2A (PRRC2A) reportedly binds m^6^A-modified mRNAs to regulate oligodendrocyte differentiation and central nervous system function. Studies have shown that PRRC2A affects mRNA stability and translation efficiency, highlighting its impact on cellular development (45, 46).

Building upon the core machinery of writers, erasers, and readers, it is increasingly recognized that the regulatory apparatus of m^6^A modification itself operates within a complex epigenetic landscape. Emerging evidence indicates that these m^6^A regulators are subject to diverse post-translational modifications, including ubiquitination, SUMOylation, acetylation, phosphorylation, O-GlcNAcylation, ISGylation, and lactylation, which finely modulate their activity and stability in cancer contexts (47). Besides, noncoding RNAs have been implicated in regulating the activity of the m^6^A machinery. This higher-order layer of epigenetic control adds significant complexity to m^6^A-mediated RNA metabolism and underscores the need to further investigate how such regulatory mechanisms contribute to prostate-related disease pathogenesis. Elucidating these interactions could reveal novel therapeutic vulnerabilities for precision oncology (47).

## 4 The regulatory role of m^6^A methylation in prostatitis

The core pathological mechanism of chronic prostatitis involves an imbalance between tissue damage and repair caused by repeated inflammatory stimuli. Inflammatory cells infiltrate prostate tissue, release pro-inflammatory factors such as tumor necrosis factor-alpha (TNF-α) and IL-1β, and induce local microenvironment disorder and acinar epithelial damage (48). In cases of chronic, unresolved inflammation, this process is closely related to abnormal glycolysis and immune cell polarization (49). Although direct empirical evidence characterizing m^6^A methylation in prostatitis remains sparse, emerging indirect data suggest a significant associative link.

### 4.1 The role of m^6^A methylation in the regulation of inflammation

m^6^A methylation modification plays a vital role in the regulation of inflammation. Studies have shown that WTAP, as the core component of the m^6^A MTC, is directly regulated by NF-κB p65, forming a positive feedback loop that promotes the inflammatory response (50).

In terms of molecular mechanisms, WTAP promotes MTC aggregation in the nucleus via phase separation, enhances m^6^A modification of inflammation-related transcripts (such as IL-6, TNF-α), and subsequently amplifies pro-inflammatory signals by regulating mRNA stability. Experiments have confirmed that myeloid cell-specific knockout of WTAP could significantly alleviate LPS-induced sepsis and DSS-induced colitis symptoms (50). These findings suggest that the WTAP-m^6^A axis may serve as a potential therapeutic target for inflammatory diseases.

### 4.2 m^6^A methylation as a common regulatory mechanism in organ fibrosis

Current evidence suggests that m^6^A methylation exhibits a common regulatory mechanism in organ fibrosis. Studies have shown that the MTC (METTL3, METTL14, WTAP) is involved in the fibrotic process by mediating m^6^A modification of specific RNAs (51).

In renal fibrosis, the complex promotes transforming growth factor-beta 1 (TGF-β1) signaling pathway activation by upregulating miR-21, and an abnormal m^6^A modification pattern can lead to dysregulated expression of fibrosis-related genes such as *SLC4A1* and *THY1* (51). This mechanism is conserved among organs. For example, METTL3 regulates FoxO1 mRNA through m^6^A modification in pulmonary fibrosis to affect vascular endothelial cell function (52), while liver fibrosis relies on METTL3-mediated Hippo/YAP signaling pathway to activate hepatic stellate cells (53). Notably, TGF-β1 is a core mediator of fibrosis, and m^6^A-dependent regulation of its signaling pathway may constitute a common molecular basis for fibrosis across organs. These findings suggest that targeting m^6^A-modifying enzymes may become a general strategy to reverse fibrosis.

### 4.3 m^6^A methylation as a putative regulator in prostatitis

Prostatitis is a critical initiating factor in the prostatitis-BPH-PCa disease continuum, in which chronic inflammation driven by prostatitis leads to sustained NF-κB activation and the release of IL-6, IL-8, TNF-α, and TGF-β. These cytokines induce oxidative stress, DNA damage, and genomic instability, creating a pro-tumorigenic microenvironment that primes the prostate for malignant transformation (2, 13). This inflammatory milieu facilitates progression from benign conditions to cancer through persistent signaling and molecular alterations.

Although direct research on m^6^A modification in prostatitis remains limited, insights from PCa and other fibrotic diseases provide cross-system evidence. In PCa, various RNA modifications, including m^6^A, play crucial roles in modulating AR signaling and tumor progression. For instance, key m^6^A regulators such as METTL3 and ALKBH5 have been shown to influence AR activity. METTL3 promotes AR expression and the activation of its target genes by facilitating m^6^A methylation on relevant transcripts. Conversely, ALKBH5 stabilizes SIAH1 mRNA, which can suppress the expression of AR splice variants such as AR-v7 (54). These findings highlight the intricate regulatory mechanisms of RNA modifications in shaping the cellular fate and metabolism in PCa. The m^6^A reader HNRNPC drives immunosuppression by promoting Treg activation and inhibiting CD8^+^ T cell function, a mechanism potentially relevant to chronic prostatitis progression (55). Furthermore, the METTL3/m^6^A/Smad3 axis identified in renal fibrosis models highlights how m^6^A regulates TGF-β1 signaling and extracellular matrix (ECM) deposition via pri-miR-21 maturation and Smad3 mRNA stability (56, 57). Given the conserved role of TGF-β1 in multi-organ fibrosis, this pathway likely contributes to prostate tissue remodeling and fibrotic progression in prostatitis.

Collectively, these findings suggest that m^6^A methylation may represent a shared molecular mechanism linking prostatitis to fibrosis and carcinogenesis. By modulating AR signaling, immune microenvironment, and profibrotic pathways, m^6^A modification offers a promising framework for targeted interventions in prostatitis.

## 5 m^6^A modification and BPH

In recent years, the role of epigenetic regulation in prostate-related diseases has gradually become a research hotspot. Indeed, RNA m^6^A modification, as a dynamic and reversible post-transcriptional modification mechanism, plays an essential regulatory role in tumorigenesis, tissue fibrosis, and other processes (18, 58). BPH, as the most common urinary system disease in elderly men, is characterized by an imbalance between epithelial mesenchymal cell proliferation and apoptosis (59). Although previous studies have shown that DNA methylation and histone modification are involved in the pathogenesis of BPH, the specific mechanism of m^6^A modification in BPH remains unclear (60).

### 5.1 m^6^A methylation-mediated mechanisms and functional regulation in BPH pathogenesis

BPH represents a critical stage within the prostatitis-BPH-PCa pathological continuum. Chronic inflammation-driven tissue remodeling, including EMT and dysregulation of the AR signaling axis, fosters a microenvironment conducive to the development of pre-cancerous lesions (e.g., PCa and high-grade prostatic intraepithelial neoplasia) and potential malignant transformation. Accumulating evidence implicates aberrant m^6^A RNA methylation as a pivotal mechanism promoting this progression (2, 9, 13).

Given the shared pathological underpinnings of chronic inflammation and AR signaling dysregulation between BPH and PCa, insights from the more extensively studied m^6^A landscape in PCa offer a rational framework for elucidating parallel mechanisms in BPH (2, 10, 13). This heterogeneity underscores the dynamic regulation of the post-transcriptional landscape by m^6^A in driving BPH pathogenesis. The core machinery of m^6^A modification is deeply involved in this pathological remodeling. Current evidence suggests that the expression of the m^6^A methyltransferase METTL3 is significantly upregulated in hyperplastic tissues. Functionally, METTL3 promotes prostatic hyperplasia by regulating PTEN expression in an m^6^A-YTHDF2-dependent manner. Specifically, METTL3 increases the m^6^A modification of PTEN and inhibits its expression through the reading protein YTHDF2. This mechanism disturbs the balance between epithelial proliferation and apoptosis, promotes epithelial-mesenchymal transition (EMT), and accelerates BPH development. Therefore, the METTL3/YTHDF2/PTEN axis plays a crucial role in the pathogenesis of BPH (61, 62). Conversely, reader proteins exert fine-tuned control; for instance, YTHDC2, a protein with RNA helicase activity, can regulate RNA structure and stability. In PCa metastasis, YTHDC2 expression is abnormally elevated, which may enhance the invasion and metastasis ability of cancer cells (34). These dysregulated m^6^A modifications critically impair fundamental cellular processes in BPH. A pivotal mechanism involves the regulation of EMT. Specifically, studies in prostate and other urological cancers demonstrate that METTL3-mediated m6A modification of ZEB1 mRNA enhances its binding to the reader protein YTHDF2, promoting ZEB1 mRNA degradation and thereby exerting dynamic control over EMT—a process modulated by TGF-β concentration gradients within the microenvironment (63). Furthermore, evidence from gastrointestinal and pulmonary epithelial pathologies indicates that METTL14 influences proliferation pathways, such as EGFR signaling (64), suggesting that analogous regulatory axes may contribute to stromal dysregulation in BPH pathogenesis.

Overall, m^6^A serves not merely as an independent epigenetic mark but also as a crucial regulatory node that integrates diverse pathological stimuli, including hormonal signals, inflammatory factors, and mechanical stress, in BPH. Given its instrumental role in regulating key processes along the BPH-PCa continuum, targeting m^6^A methylation emerges as a promising therapeutic strategy for BPH intervention and potentially for mitigating disease progression (9, 13, 63).

## 6 Dysregulation of m^6^A regulators in PCa

Building on the core machinery of m^6^A regulators detailed in Section 3, this section examines their specific dysregulation and pathogenic roles in PCa. Aberrant expression and activity of these regulators drive PCa initiation, progression, metastasis, and therapy resistance by rewiring the post-transcriptional landscape of key oncogenes and tumor suppressors. The dynamic interplay between writers, erasers, and readers orchestrates a complex regulatory network that contributes significantly to disease heterogeneity and clinical outcomes.

### 6.1 MTC

#### 6.1.1 METTL3

In PCa, METTL3 expression is significantly upregulated in cancer cell lines and clinical specimens, and correlated with tumor malignancy (69). Functionally, METTL3 promotes oncogenesis by targeting a repertoire of transcripts. It enhances the stability of kinesin superfamily member 3 (KIF3) mRNA through m^6^A modification, thereby accelerating cancer cell proliferation, migration, and invasion (65). Yuan et al. (66) further revealed the key role of the METTL3-MYC regulatory axis: upregulated m^6^A level of MYC mRNA in cancer tissues was significantly correlated with METTL3 overexpression, and wild-type METTL3 overexpression could specifically enhance MYC protein translation, confirming that its oncogenic effect is dependent on methyltransferase activity.

Recent advancements in molecular profiling have facilitated the stratification of PCa into five distinct m^6^A-driven subtypes (P1-P5). Notably, the P3 cohort is characterized by a robust enrichment of METTL3 and YTHDF2 expression, which is significantly associated with tumor aggressiveness and postoperative recurrence (16). In addition, METTL3 regulates tumor metabolism through the IGFBP3/AKT pathway, and its small-molecule inhibitor STM2457 can reduce m^6^A levels and inhibit cancer cell stemness, showing synergistic efficacy in preclinical models (67). Notably, METTL3-mediated m^6^A modification of the *VCAN* gene under hypoxia stabilizes mRNA through IGF2BP protein and promotes tumor metastasis. This mechanism has been validated in 75% of bone metastasis cases (16). These findings not only confirm the core position of the METTL3-m^6^A-MYC axis, but also reveal its complex association with tumor heterogeneity, metabolic adaptation and microenvironment remodeling, providing novel insights for the development of hierarchical diagnosis and treatment strategies based on m^6^A epigenomics.

#### 6.1.2 METTL14

In PCa, METTL14 plays a critical role in promoting tumorigenesis by regulating key downstream targets. A prime mechanism involves the suppression of Thrombospondin 1 (THBS1), a natural inhibitor of angiogenesis. METTL14 reduces THBS1 expression by binding to its 3’ untranslated region and facilitating its m^6^A-dependent degradation, primarily through the reader protein YTHDF2, thereby promoting PCa development by enhancing angiogenesis (68). This mechanism was further verified in a multicenter study published in 2025. MeRIP-seq analysis found that high METTL14 expression in 162 PCa samples was significantly correlated with low THBS1 expression, and this negative correlation was particularly prominent in tumors exhibiting aggressive histological features, specifically those with the intraductal carcinoma and cribriform architecture (IDC/CA) subtype (16, 69).

From the perspective of molecular mechanisms, METTL14-mediated m^6^A modification of THBS1 mRNA mainly occurs at GGAC motif sites, and YTHDF2 specifically recognizes these modification sites through its YTH domain, which shortens the half-life of THBS1 mRNA by about 60%, finally leading to the decline of THBS1 protein level (68). In terms of clinical significance, the dysregulation of the METTL14-THBS1 axis is closely related to the poor prognosis of PCa. TCGA database analysis showed that the 5-year survival rate of patients with high METTL14 expression/low THBS1 expression was only 34.7%, significantly lower than that of the control group (62.1%) (68, 69). Considerable advancements have been achieved in the development of pharmacological strategies aimed at disrupting this specific pathway. STM2457, a small molecule inhibitor identified in 2024, can reportedly indirectly restore THBS1 expression by inhibiting the function of METTL3/METTL14 complex and reduce tumor microvessel density by 41% in PDX model (67). These findings not only reveal a new mechanism by which m^6^A modification regulates tumor angiogenesis, but also provide a potential target for the development of combined anti-angiogenic therapies.

#### 6.1.3 VIRMA

VIRMA plays a key oncogenic role in PCa. A recent multi-omics study revealed that VIRMA expression was significantly elevated in hormone-insensitive PCa, and its copy number amplification was positively correlated with patients’ risk of biochemical recurrence. Single-cell sequencing showed that it was specifically enriched in tumor stem-like cell subsets (68). In terms of molecular mechanisms, VIRMA promotes tumor progression primarily by globally regulating m^6^A epigenome homeostasis: its knockdown reduces m^6^A modification by 40-60%, destabilizing oncogenic lncRNAs (e.g., PCaT1, MALAT1). Besides, VIRMA specifically targets VCAN mRNA via m^6^A modification, extending its half-life through the YTHDF1-IGF2BP axis to promote extracellular matrix remodeling and metastasis (16). Clinical analysis showed that the high-expression subtype of VIRMA (P3 subtype) exhibited significant hypoxic characteristics and was strongly correlated with the aggressive histological subtype. Importantly, the hypoxic microenvironment could further induce VIRMA expression, forming a positive feedback loop (16, 70). In terms of treatment, VIRMA targeted shRNA combined with the PARP inhibitor olaparib could reduce xenograft tumor volume by 62%, suggesting its potential as a combined therapeutic target (67).

Beyond the intrinsic catalytic functions of individual m^6^A regulators, their interplay with canonical oncogenic pathways critically shapes PCa pathogenesis. The crosstalk between m^6^A modification and AR signaling represents a pivotal axis in this regard. As comprehensively reviewed by Cao et al., AR remains the central driver of PCa progression, with emerging evidence suggesting that m^6^A signaling, through its regulators (writers, erasers, and readers), interacts with AR pathways to influence disease development (71). Given that both AR-dependent and AR-independent mechanisms contribute to castration resistance, understanding how m^6^A regulators modulate these networks is essential for advancing therapeutic strategies in advanced PCa (71). This interplay provides a foundational framework for understanding the specific roles of the erasers and readers detailed in the following sections.

### 6.2 Demethylases

#### 6.2.1 FTO

As a tumor suppressor in PCa, FTO expression is significantly downregulated in PCa tissues and correlates with adverse clinical features such as high tumor stage and Gleason score (72). Functional assays have demonstrated that FTO depletion promotes PCa cell metastasis and invasion through multiple mechanisms, including: 1) m^6^A-dependent regulation of Melanocortin 4 Receptor (MC4R) expression (73); 2) maintenance of Chloride Channel Protein 4 (CLIC4) mRNA stability (15); 3) the targeted regulation of DDIT4 mRNA (74). Furthermore, tumor hypoxic microenvironments can remodel FTO activity, with hypoxia-induced FTO suppression exacerbating m^6^A imbalance and promoting PCa progression (16). Notably, a 2024 study revealed that the transcription factor ZFHX3 could inhibit FTO expression by directly binding to the FTO promoter, thereby increasing the level of m^6^A modification and inhibiting the proliferation of PCa cells, providing new insights into the upstream regulatory mechanism of FTO (75). In addition, preclinical studies have shown that small-molecule inhibitors targeting FTO (such as FB23-2) can inhibit PCa cell growth in a dose-dependent manner (75), suggesting that FTO, as a key regulator of m^6^A modification, represents a potential target for PCa treatment.

#### 6.2.2 ALKBH5

In PCa, the expression of the m^6^A demethylase ALKBH5 is significantly downregulated and negatively correlated with miR-141-3p expression (76). Functionally, Upstream Stimulator Factor 1 (USF1) exerts a tumor-suppressive role by transcriptionally activating ALKBH5, which in turn enhances the stability of Flightless-I (FLII) mRNA in a YTHDF2-dependent manner, thereby inhibiting glycolytic activity, proliferation, and metastasis of PCa cells (77).

In addition, a 2025 study further revealed the complex regulatory network of m6A modification in PCa, finding that ALKBH5 expression is closely associated with the tumor hypoxic microenvironment. Hypoxia was shown to induce the downregulation of ALKBH5, thereby forming a positive feedback loop that promotes tumor progression (16). Preclinical studies have also shown that small-molecule inhibitors targeting ALKBH5 can significantly inhibit the proliferation and migration of PCa cells, suggesting its potential value as a therapeutic target (16). These findings provide a new perspective for understanding the role of m^6^A modification in the occurrence and development of PCa and lay a theoretical foundation for the development of therapeutic strategies based on m^6^A regulation.

### 6.3 Methylation reader

#### 6.3.1 YTHDF family

Members of the YTHDF family are critically involved in PCa progression through distinct mechanisms. YTHDF1 is highly expressed in PCa and promotes translation of RNF7 mRNA via an m^6^A-dependent mechanism, thereby accelerating ubiquitination and degradation of p27, a cell cycle inhibitor, and promoting tumor cell proliferation (78). Clinical data showed that high expression of YTHDF1 was significantly associated with poor prognosis of patients, and knockdown of YTHDF1 could significantly enhance the sensitivity of PCa cells to cisplatin and other chemotherapeutic drugs (79).

YTHDF2 is also overexpressed in PCa, which mediates the degradation of tumor suppressor NK3 Homeobox 1 (NKX3-1) and Phospholysine Phosphohistidine Inorganic Pyrophosphate Phosphatase (LHPP) mRNA through an m^6^A-dependent manner, and then activates the Akt signaling pathway to promote tumor migration (17). The study further revealed that high YTHDF2 expression was significantly associated with the aggressive histological subtype of PCa (IDC/CA) and was closely related to the tumor microenvironment’s hypoxia (16).

YTHDF3 works synergistically with GTPase-activating protein binding protein 1 (G3BP1) to regulate AR mRNA translation: m^6^A-modified AR mRNA is activated after binding with YTHDF3, while unmodified AR mRNA is inhibited after binding with G3BP1 (16). Notably, the study found that YTHDF3 silencing can significantly improve the sensitivity of PCa cells to AR Pathway Inhibitors (ARPI) and reduce the xenograft tumor volume by 62% (80).

In addition, a recent study found that YTHDC2 is specifically overexpressed in PCa stem cells and promotes tumor stemness maintenance by regulating the Wnt/β-catenin signaling pathway (16). These findings suggest that YTHDF family members synergistically promote PCa progression through distinct molecular mechanisms and may serve as potential targets for combination therapy.

#### 6.3.2 IGF2BP

The IGF2BP family plays a significant role in PCa pathogenesis, especially in aggressive and metastatic disease. A key example is the interaction between IGF2BP2 and the lncRNA PCaT6, which is markedly upregulated in bone metastatic tissues and correlates with poor prognosis. Mechanistically, PCaT6 recruits IGF2BP2 to stabilize its own m^6^A modifications, thereby enhancing Insulin-like Growth Factor 1 Receptor (IGF1R) mRNA translation and activating the PI3K/AKT signaling pathway to drive PCa cell proliferation and bone metastasis (16). This axis also promotes tumor metabolic adaptability by upregulating BCAT2, associated with high-risk malignancies (81). Notably, IGF2BP3-mediated stabilization of JPX may interact with GOLM1, a diagnostic marker for PCa patients in the PSA “gray zone”, highlighting its potential clinical utility (81). Furthermore, considering the unique molecular characteristics of PCa in Asian populations, particularly the specific enrichment of type 2 luminal cells, the IGF2BP family emerges as a promising target for precision medicine approaches tailored to regional epidemiological patterns (82). Current evidence positions the IGF2BP family as a critical regulator of multiple oncogenic processes in PCa, including growth, metastasis, metabolic reprogramming, and treatment resistance. Through their interactions with m^6^A-modified lncRNAs and other RNA species, IGF2BP proteins bridge molecular alterations to clinically relevant features, such as East-West genomic differences and diagnostic challenges in PSA interpretation. Future research should integrate multi-omics data to fully characterize the clinical and translational potential of IGF2BP proteins and facilitate the development of personalized therapeutic strategies.

#### 6.3.3 hnRNP

hnRNPs are significantly involved in PCa progression. HNRNPA2B1 expression is upregulated in PCa and enhances tumor cell invasiveness by promoting the maturation of oncogenic miRNAs, particularly miR-21 (83). Multi-omics studies have demonstrated that its upregulation is correlated with aggressive clinicopathological features (Gleason score >7, pT3 stage, TP53 mutations) and serves as a predictor for biochemical recurrence risk (16). Mechanistically, HNRNPA2B1 regulates the mRNA stability of key oncogenes like *MYC* and *AR* through m^6^A modification recognition (84).

Functional studies have revealed that HNRNPH/F double knockout significantly downregulates MYC expression, increases cleaved PARP levels, and induces apoptosis in PC3 cells, while causing a reduction in G1 phase and a G2 phase arrest, thereby inhibiting mitotic progression (85). The m^6^A modification peak of the HNRNPLL gene has been found to correlate with tumor hypoxic microenvironment, suggesting hnRNPs may regulate m^6^A modifications in response to environmental signals (16). Although the specific mechanisms of HNRNPC and HNRNPG in PCa require further investigation (86, 87), existing evidence indicates that HNRNPC participates in m^6^A-dependent alternative splicing regulatory networks (84). Recent reviews, including work by Zhang Qian’s team at Peking University, have emphasized the hnRNP family’s potential as therapeutic targets, particularly for treatment-resistant PCa cases (16). These findings collectively demonstrate that hnRNPs exert multi-level regulatory functions in PCa development by integrating the m^6^A epigenome regulatory network, making them promising candidates for future targeted therapies.

#### 6.3.4 Other m^6^A reader proteins in PCa

Beyond the well-characterized families, several other RNA-binding proteins may function as m^6^A readers in PCa. eIF3 contributes to castration-resistant prostate cancer (CRPC) progression by mediating non-canonical translation pathways that may facilitate tumor cell adaptive survival (88).

**Figure 3:**
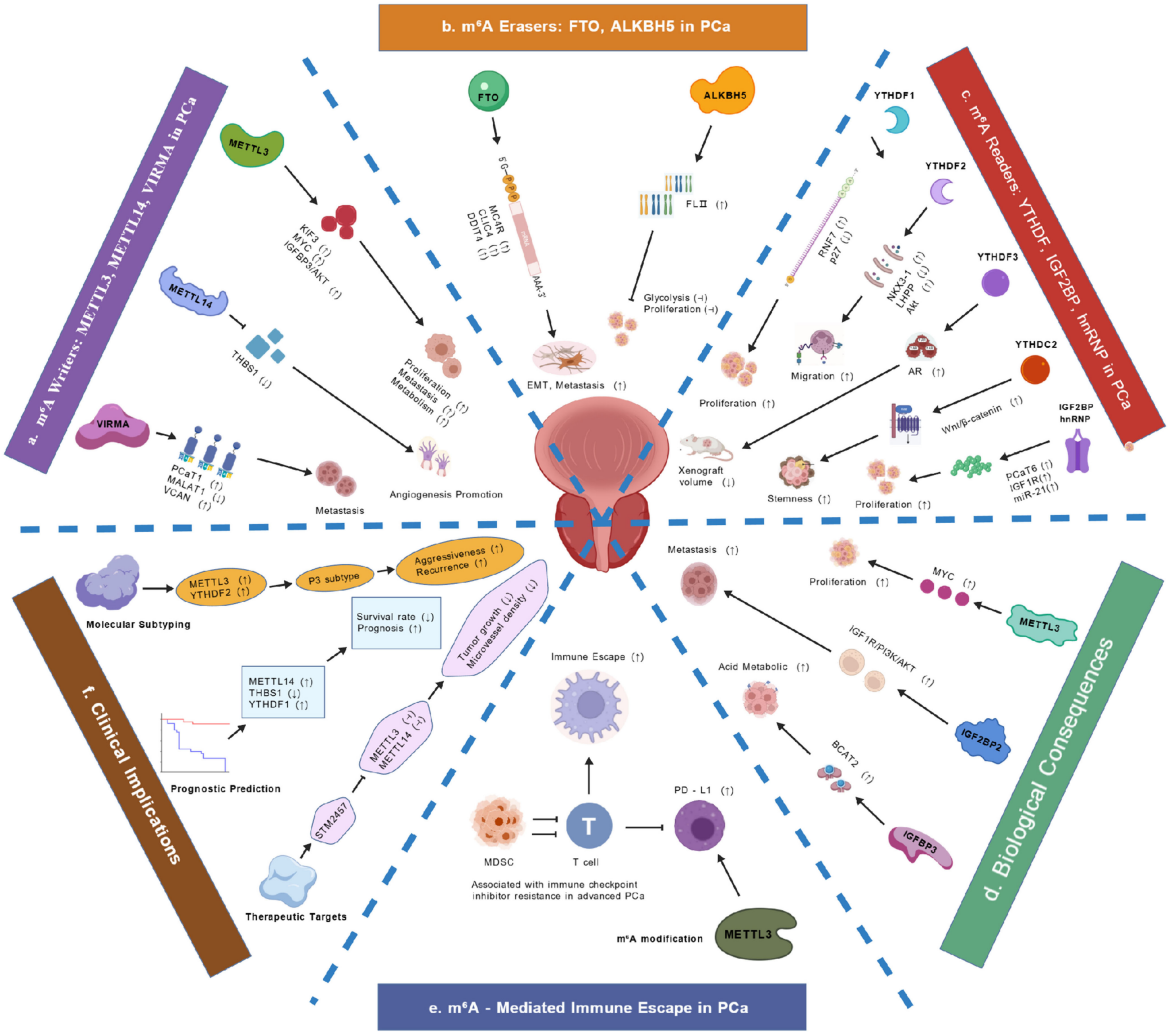
Schematic network of m^6^A methylation regulators in PCa: mechanisms, biological effects, and clinical implications. This figure illustrates the integrated regulatory network of m^6^A methylation in PCa, highlighting the roles of writers, erasers, readers, and their downstream effects on tumorigenesis, progression, immune evasion, and clinical outcomes. The MTC, including METTL3, METTL14, and VIRMA, catalyzes m^6^A deposition, promoting tumor proliferation, angiogenesis, and metastasis. METTL3 stabilizes oncogenic transcripts such as MYC and VCAN, while METTL14 downregulates thrombospondin 1 (THBS1) via m^6^A-mediated degradation, enhancing angiogenesis. VIRMA amplifies m^6^A signaling in aggressive subtypes (e.g., P3), driving stemness and hypoxia adaptation. Demethylases FTO and ALKBH5 counteract m^6^A marks; FTO loss promotes EMT and glycolysis, while ALKBH5 depletion enhances glycolytic activity and tumor growth. Readers, including YTHDF1–3, IGF2BP family, and hnRNPs, interpret m^6^A modifications to regulate mRNA stability, translation, and splicing. YTHDF1 enhances RNF7 translation to degrade p27, YTHDF2 degrades NKX3–1 and LHPP to activate Akt, and YTHDF3 modulates AR signaling. IGF2BP proteins stabilize oncogenic lncRNAs (e.g., PCaT6) and promote PI3K/AKT activation and bone metastasis. HNRNPA2B1 regulates MYC and AR stability, contributing to aggressiveness. m^6^A modification also facilitates immune escape by upregulating PD-L1 expression on tumor cells, suppressing T-cell function and promoting immunosuppressive microenvironments. Clinically, m^6^A regulators correlate with Gleason score, molecular subtypes (e.g., P3), biochemical recurrence, and treatment resistance. High METTL3/YTHDF2 defines an aggressive subtype associated with poor survival. Targeting the m^6^A machinery, such as STM2457 inhibition of METTL3/METTL14 or PARP inhibitor combination with VIRMA knockdown, shows therapeutic potential. This network underscores the central role of m^6^A in PCa pathogenesis and highlights its value for prognostic prediction and precision therapy.

Although the role of FMRP in CRPC progression has not been conclusively established, it exhibits oncogenic potential, as evidenced by its upregulation in multiple cancers and potential involvement in tumor immune evasion mechanisms (89, 90). These findings suggest FMRP may influence PCa metastasis, particularly through MET regulation, though this requires further experimental validation. Current evidence does not sufficiently support a direct role for PRRC2A in PCa progression (45, 46), highlighting the need for additional investigation into its potential functions in m^6^A-mediated oncogenic processes. The distinct and potential mechanisms of these non-canonical m^6^A readers in PCa are summarized and illustrated in **Figure 3**.

### 6.4 m^6^A modification and immune microenvironment of PCa

The Tumor Immune Microenvironment (TIME) is a complex ecosystem of immune cells, stromal cells, signaling molecules, and non-cellular components that surrounds tumor cells. It is a critical determinant of tumor fate, profoundly influencing cancer progression, metastasis, and response to therapy. In PCa, the TIME is typically characterized as “immunologically cold”, exhibiting features such as low T-cell infiltration, an abundance of immunosuppressive cells (e.g., Tregs, M2 macrophages), and upregulated immune checkpoint molecules, which collectively contribute to the limited efficacy of immunotherapies (91–93). Building on this, the immunomodulatory role of m^6^A extends to shaping the TIME, with growing relevance to immunotherapy in PCa. As highlighted by Han et al., m^6^A modification is involved in PCa pathogenesis and progression, and its potential connection to immunotherapy is increasingly recognized (94). m^6^A-based targets offer new avenues for PCa treatment, including strategies that leverage the interplay between RNA epigenetics and immune responses. While clinical trials are exploring m^6^A-related approaches, further research is needed to fully elucidate the underlying mechanisms and therapeutic potential (94).

Beyond their cell-intrinsic oncogenic roles, m^6^A modifications extend their influence to the TIME of PCa through three interconnected mechanisms: (1) direct regulation of immune checkpoint molecules (e.g., PD-L1); (2) modulation of cytokine and chemokine networks that govern immune cell recruitment and polarization; and (3) alteration of the functional state of tumor-infiltrating immune cells themselves, such as T cells, macrophages, and myeloid-derived suppressor cells (MDSCs). The following sections will detail how individual m^6^A regulators, including writers, erasers, and readers, exert these immunomodulatory functions to foster an immunosuppressive niche.

The dynamic remodeling of the TIME is orchestrated by intricate molecular networks, among which m^6^A RNA methylation has emerged as a pivotal layer of post-transcriptional regulation. The functional impact of m^6^A modification on the PCa TIME is mediated through the coordinated and often antagonistic actions of its regulatory machinery—writers (methyltransferases), erasers (demethylases), and readers (recognition proteins). The methyltransferase METTL3, a core writer, predominantly fosters an immunosuppressive niche. METTL3 deficiency in tumor cells facilitates the recruitment of tumor-associated macrophages (TAMs) and regulatory T cells (Tregs), while the METTL3/YTHDF2 axis augments immunosuppression by tumor-infiltrating myeloid cells via the JAK1/STAT1 pathway, promoting immune evasion (95, 96). Conversely, the pharmacological inhibition of demethylases (erasers), such as with meclofenamic acid, can produce an opposing, immunostimulatory effect by promoting immunogenic cell death and activating dendritic and effector T cells (97). This yin-yang of immune regulation is further interpreted by reader proteins. Among m^6^A reader proteins, HNRNPA2B1 exerts potent immunosuppressive effects in PCa by promoting PD-L1 expression, thereby facilitating immune escape and driving castration-resistant progression (98). Notably, emerging evidence also implicates HNRNPA2B1 in modulating Treg dynamics within the TIME, although the precise molecular intermediaries remain under investigation. This aligned with the broader role of hnRNP family readers in shaping an immune-cold landscape: for example, HNRNPC has been shown to actively suppress antitumor immunity in immunologically “cold” PCa subtypes by promoting Treg activation and inhibiting effector CD8^+^ T-cell function (55). Furthermore, the functional balance of T-cells is fine-tuned by m^6^A, as evidenced by METTL14 and YTHDF2 in macrophages modulating T-cell phenotypes, with METTL14 deletion exacerbating CD8^+^ T-cell exhaustion (99).

This comprehensive understanding positions the m^6^A modification landscape as a master regulator of the immunosuppressive phenotype in PCa. Computational models, such as the m^6^A scoring system, demonstrate that low m^6^A scores correlate with improved immunotherapy response and prognosis, directly linking the m^6^A regulatory patterns to TIME cell infiltration and immune-related pathways (100, 101). Therefore, targeting specific m^6^A regulators presents a promising strategy to reprogram the “cold” TIME of PCa into an immune-responsive “hot” state, thereby overcoming the current limitations of immunotherapy. Future efforts should focus on validating these mechanisms in clinically annotated patient cohorts to translate these insights into effective combination immunotherapies.

## 7 The diagnostic and therapeutic potential of m^6^A methylation in prostate-related diseases

m^6^A methylation modification demonstrates significant diagnostic and therapeutic potential in prostate-related diseases. As a diagnostic tool, m^6^A modification patterns show potential as sensitive and specific biomarkers for PCa. Studies have revealed that abnormal expression of m^6^A-modifying enzymes (such as METTL3 and FTO) is significantly correlated with tumor grade and metastatic risk (102). Through MeRIP-seq analysis of 162 PCa samples, five m^6^A molecular subtypes were identified, which showed significant associations with tumor aggressiveness, genomic instability, and postoperative recurrence risk. Notably, m^6^A modification of the VCAN gene promotes tumor invasion by stabilizing mRNA through IGF2BP, and its expression level can predict biochemical recurrence risk. Furthermore, alterations in the activity of m^6^A regulatory factors (such as ALKBH5) in serum or urine may serve as non-invasive diagnostic tools (16).

The diagnostic potential of m^6^A methylation provides a rationale for developing targeted therapeutic strategies against this epigenetic pathway. Current investigative agents can be broadly categorized into synthetic small-molecule inhibitors and natural compounds, each demonstrating unique mechanisms of action at various stages of preclinical and clinical development. Among synthetic small-molecule inhibitors, targeted suppression of m^6^A “writers” and “erasers” has demonstrated significant preclinical promise. Evidence from *in vitro* cell culture and *in vivo* xenograft models substantiates that the METTL3 inhibitor STM2457 effectively inhibits the proliferation, migration, and invasion of PCa cells via the IGFBP3/AKT pathway (67). Similarly, inhibitors targeting the demethylases FTO (e.g., FB23-2) and ALKBH5, primarily validated in preclinical models, can reverse m^6^A dysregulation, particularly in hypoxic microenvironments, presenting opportunities to overcome therapy resistance (16). Furthermore, *in vitro* spectroscopy and molecular docking studies indicate that camptothecin analogs can directly bind and inhibit FTO (103), providing a mechanistic rationale for their future investigation.

Natural products constitute another major class of m^6^A modulators, with evidence ranging from mechanistic *in vitro* studies to clinical trials confirming bioavailability and preliminary efficacy. The anti-tumor effects of curcumin are supported by a multi-level evidence chain: *In vitro* studies demonstrate its inhibition of METTL3, which reduces m^6^A modification on oncogenic circular RNA (circRNA)s (e.g., circ0030568) and leads to their destabilization (104). Crucially, these mechanistic findings are complemented by clinical evidence from a randomized controlled trial in patients, in which curcumin supplementation significantly lowered the rate of PSA progression in men undergoing intermittent androgen deprivation therapy (1). Other natural compounds, including epigallocatechin gallate (EGCG) and quercetin, have been shown in *in vitro* studies to modulate m^6^A regulators such as FTO and METTL3 (105). Importantly, a prospective randomized trial in men with PCa confirmed that quercetin supplementation achieves significant bioavailability in plasma, urine, and prostate tissue (106), providing a critical bridge between *in vitro* activity and potential *in vivo* efficacy.

Notably, several m^6^A-targeting strategies exhibit enhanced efficacy when combined with established therapies, with evidence from diverse experimental settings. m^6^A modulation can produce synergistic effects with PARP inhibitors or immunotherapies, as suggested by studies in preclinical models (67, 107). Simvastatin has also emerged from repurposing screens as a candidate, with its METTL3-downregulating activity, first identified in lung cancer (108), now shown to be relevant in PCa. Studies combining CRPC patient sample analysis with *in vitro* validation demonstrate that simvastatin can synergize with AR antagonists (109). These findings provide a rationale for its evaluation in ongoing clinical trials.

Looking forward, emerging RNA-targeting platforms represent a novel therapeutic frontier. As reviewed by Xu et al., cutting-edge approaches such as CRISPR-Cas13 systems are being explored to precisely target m^6^A regulatory pathways (110). This strategy aims to modulate the activity of writers, erasers, and readers to disrupt oncogenic RNA modification networks. While these platforms hold significant promise for developing novel therapies in PCa, critical challenges regarding specificity and delivery efficiency must be addressed before clinical translation (110).

In summary, the strategic inhibition or modulation of m^6^A regulators offers a novel and multifaceted approach to PCa treatment. The main classes of m^6^A-targeting agents, their molecular targets, and the supporting evidence across the full translational spectrum, from mechanistic studies and preclinical models to clinical trials, are systematically summarized in **Table 1**. Future research should prioritize the clinical validation of these therapeutic strategies, particularly advancing agents with strong *in vitro* and *in vivo* preclinical data into well-designed human trials.

**Table 1:** Research progress of m^6^A-related diagnostic and therapeutic indicators in PCa.

Category	Indicator/drug	Target	Research type	Research phase	Country	Primary outcome	References
Diagnostic Indicators	m^6^A modification enzyme profile	METTL3/FTO etc.	Clinical studies	Clinical validation	Multinational	Tumor grade prediction	(117)
	VCAN m^6^A modification	IGF2BP	Clinical studies	Clinical validation	Multinational	Biochemical recurrence prediction	(16)
	ALKBH5 activity	ALKBH5	Clinical studies	Clinical validation	Multinational	Non-invasive diagnosis	(16)
Therapeutic Drugs	STM2457	METTL3	Preclinical models	Preclinical	Laboratory	Tumor proliferation inhibition	(67)
	FB23-2	FTO	Preclinical models	Preclinical	Laboratory	Hypoxic microenvironment reversal	(16)
	Curcumin	ALKBH5/YTHDF1	*In vitro* & Clinical	Phase 3 (NCT03769766)	United States	Disease progression rate	(1, 118)
	Simvastatin	METTL3	Cell/Patient data & Clinical Trial	Phase 2 (NCT05586360)	United States	YAP-mediated Tregs dysfunction in prostate	(108, 109)
	EGCG	FTO/YTHDF2	*In vitro* studies	Phase 2 (NCT04300855)	United States	Disease progression rate	(119)
	Quercetin	METTL3	*In vitro* & Clinical	Clinical evaluation	Multinational	Tumor microenvironment modulation	(105, 106)
	Camptothecin analogs	FTO	*In vitro* studies	Phase1-2 (NCT02769962)	United States	Objective response rate	(103)

## 8 Challenges

m^6^A methylation, as a key mechanism of epigenetic regulation, plays an important role in prostate-related diseases, including prostatitis, BPH, and PCa. In recent years, with the deepening of high-throughput sequencing technology and epigenetic research, the regulatory network of m^6^A modification in prostate-related diseases has gradually become clear. As a systematic comparison in **Table 2** illustrates, the specific mechanisms and pathophysiological consequences of m^6^A dysregulation exhibit both shared themes and distinct features across prostatitis, BPH, and PCa. However, there are still significant differences in its specific mechanism of action and clinical translational potential in different diseases.

**Table 2:** Integrated summary of similarities and differences in m^6^A methylation across prostatitis, BPH, and PCa.

Key similarities	Key differences
Core pathological link: Chronic inflammation and androgen receptor (AR) signaling dysregulation represent a common pathological basis across the disease spectrum. m^6^A methylation acts as a key epigenetic modifier linking these stimuli to disease progression (2, 13).	Evidence base and mechanistic detail: • Prostatitis: Heavily relies on indirect evidence/cross-system extrapolation from PCa/fibrosis models (e.g., METTL3/ALKBH5 role inferred from PCa (53); fibrotic mechanisms inferred from renal studies (55, 56)). • BPH: Has direct evidence for core alterations (e.g., METTL3/METTL14 upregulation (61)) but understanding of its role in the immune microenvironment is limited. • PCa: Mechanisms are directly validated (e.g., METTL3 stabilizing MYC (65, 66); YTHDF2 degrading tumor suppressors (18).
Central role of m^6^A machinery: Key regulators, particularly the methyltransferase METTL3, are implicated in all three diseases, influencing cellular processes like proliferation and inflammation (53, 61, 65).	Primary pathogenic focus: • Prostatitis: Associated with sustained inflammation and potential fibrosis. • BPH: Characterized by tissue hyperplasia/remodeling and EMT (e.g., METTL3-mediated control of ZEB1 mRNA (63). • PCa: Driven by carcinogenesis, metastasis, and immune evasion (e.g., METTL3/YTHDF2 axis promoting immunosuppression (95, 96); HNRNPA2B1 promoting PD-L1 expression (98).
Therapeutic potential: m^6^A modification presents a potential diagnostic and therapeutic target for the entire spectrum of prostate-related diseases (9, 13, 63).	Clinical translation stage: • Prostatitis and BPH: Significant lag. Research is primarily at the basic science stage, validating targets and exploring therapeutic concepts. Prostatitis is the least studied. • PCa: Most advanced, with target identification and preclinical development of inhibitors (e.g., METTL3 inhibitor STM2457 (67).

To date, the majority of research has focused on PCa, establishing a relatively comprehensive m^6^A regulatory network within this disease context. It is now understood that that m^6^A modifying enzymes (METTL3, METTL14, WTAP complex), demethylases (FTO, ALKBH5), and reading proteins (YTHDF family, YTHDC family) are commonly expressed abnormally in PCa, and affect tumor cell proliferation, invasion, metastasis, and drug resistance by regulating key signaling pathways (PI3K-AKT, Wnt/β- catenin, AR) (107). For example, METTL3 can stabilize the mRNA of certain oncogenes such as MYC and EGFR in an m^6^A-dependent manner, promoting tumor progression. Overexpression of FTO may promote the malignant phenotype of PCa by reducing the mRNA methylation levels of tumor suppressor genes such as PTEN, thereby enhancing their degradation (15, 16). In addition, m^6^A modification regulates immune escape and metabolic reprogramming in the tumor microenvironment, providing potential targets for the development of novel immunotherapy strategies (67). As reviewed herein, the dysregulated m^6^A machinery collaborates to establish a profoundly immunosuppressive TIME, which is a major barrier to current immunotherapies. Therefore, targeting m^6^A regulators represents a novel immunomodulatory strategy. Future efforts should focus on: (1) delineating the specific immune gene repertoires controlled by each m^6^A regulator using techniques like meRIP-seq in sorted immune cells; (2) exploring combinations of m^6^A-targeting agents with immune checkpoint inhibitors to reverse the “cold” tumor phenotype; and (3) validating m^6^A-based immune signatures as biomarkers for predicting response to immunotherapy.

Compared with the relatively mature m^6^A regulatory network in PCa, research on m^6^A in BPH and prostatitis is still in its preliminary stage. In BPH, a core METTL3-PTEN axis has been established. METTL3 mediates the YTHDF2-dependent degradation of PTEN mRNA, which activates the PI3K/AKT pathway to promote epithelial proliferation and suppress oxidative stress-induced apoptosis (111). Clinical sample validation showed that PTEN protein expression in BPH tissues was significantly lower than in normal prostate tissue and was negatively correlated with m^6^A modification (16). However, direct evidence linking other m^6^A regulators and BPH remains scarce (111). Research on m^6^A modifications in prostatitis remains limited. Currently, no direct evidence verifies the hypothesis that m^6^A regulates IL-6/TNF-α or other inflammatory factors via the NF-κB pathway, nor confirms its effects on macrophage or T cell function in this context. Indirect evidence from other disease models, such as hypoxic pulmonary hypertension, suggests that low METTL3 expression may promote inflammatory factor release by reducing m6A levels (16). However, this mechanism requires validation in prostatitis through multi-omics and immune microenvironment analyses. Overall, the relationship between prostatitis and m6A represents a significant knowledge gap, underscoring the urgent need for disease-specific models and cross-omics integration studies.

In PCa, the expression levels of m^6^A regulators such as METTL3, YTHDF3, and HNRNPA2B1, as well as gene mutations (such as VIRMA), are significantly correlated with patient prognosis (10, 112). Its potential as a diagnostic or prognostic marker has been verified by multicenter cohort studies. For example, m^6^A modification of VCAN has been established as an independent prognostic marker (16). The m^6^A score molecular typing model can predict survival and treatment response in metastatic patients (10); however, its clinical applicability needs to be further confirmed by prospective clinical trials. In addition, small-molecule inhibitors targeting m^6^A-modifying enzymes, such as METTL3 inhibitor STM2457 (113) and FTO inhibitor FB23-2 (114), have shown antitumor effects in preclinical models, but their tissue specificity and safety still need to be optimized. However, at present, no m^6^A-related drugs have entered the clinical trial stage for prostate-related diseases. The main obstacles include insufficient targeting of existing inhibitors, which may cause toxicity in normal tissues, the high heterogeneity of PCa, which leads to significant differences in drug response, and the lack of reliable biomarkers to screen for sensitive patient groups. For prostatitis and BPH, the exploration of m^6^A-targeted therapy is limited, and more basic research is urgently needed to support its clinical translation. In addition, the heterogeneity of clinical samples and the lack of follow-up data also affect the clinical validation of m^6^A markers.

Indeed, progress is critically constrained by the inherent limitations of current m^6^A epitranscriptomic technologies, which directly impact the interpretation of clinical data. A primary challenge lies in the resolution and specificity of antibody-dependent detection methods. Techniques like MeRIP-seq (m^6^A RNA Immunoprecipitation Sequencing), while invaluable for transcriptome-wide profiling, possess a limited resolution that obscures the precise localization of m^6^A sites within individual transcripts. This complicates the causal linkage of a specific m^6^A modification to changes in RNA stability, splicing, or translation (115). Furthermore, antibody-based approaches can exhibit sequence bias and struggle to detect m^6^A on low-abundance transcripts, such as key transcription factors or non-coding RNA (ncRNA), which are often pivotal in disease pathogenesis. This technological gap likely leads to an underestimation of m^6^A’s regulatory scope in clinical samples. While crosslinking-based methods like miCLIP offer single-nucleotide resolution, their application to precious, often degraded, clinical archival tissues remains challenging due to technical complexity and RNA input requirements (116). The absence of robust, high-resolution m^6^A mapping tools for small clinical samples hinders the validation of putative biomarkers. Moreover, there is a pronounced lack of techniques for real-time monitoring of dynamic m^6^A changes within specific cellular compartments of the prostate tumor microenvironment, and the field urgently lacks methods for single-cell m^6^A proteomics to comprehensively identify reader, writer, and eraser proteins in individual cell types. These technological shortcomings, combined with a scarcity of animal models specific to prostate-related diseases, collectively impede a mechanistic understanding of m^6^A function in a cell-type-specific manner and its translation into reliable clinical applications.

Future research should focus on technological innovation, mechanism deepening and clinical transformation. Among them, technological innovation is the cornerstone for analyzing the dynamic regulation of m^6^A. It is necessary to develop high-sensitivity single-cell m^6^A sequencing and spatial transcriptome coupling technologies to accurately map the spatial and temporal distribution of m^6^A modification in the microenvironment of prostate tissue and to reveal the apparent transcriptome characteristics of stromal-epithelial interaction.

A more profound mechanistic understanding should prioritize the processes of benign-to-malignant transformation and their epigenetic cross-regulation. Future research must delineate the role of m6A in driving the progression from BPH to PCa and elucidate its synergistic interactions with DNA methylation and histone modifications. Translational efforts must concurrently focus on two pillars: first, the development of prostate-specific drug delivery systems, validated using advanced models such as patient-derived organoids to accurately simulate therapeutic responses; and second, fostering international multi-center collaborations to verify the clinical utility of m6A-based molecular subtyping. Accelerating the clinical translation of associated biomarkers and therapeutic inhibitors represents an urgent priority for the field.

In conclusion, while m^6^A methylation modification has broad scientific research and clinical prospects in prostate-related diseases, its full application still depends on the deepening of basic research and technological breakthroughs. In the future, it will be necessary to integrate multi-omics data, clinical resources, and new technologies to elucidate the precise regulatory mechanisms of m^6^A in prostate-related diseases and to translate these findings into precise diagnostic and therapeutic strategies.

## 9 Conclusions

m^6^A methylation functions as a master regulatory layer in prostate pathophysiology, with well-established roles in oncogenesis and emerging implications in benign prostatic diseases. While PCa research has established robust mechanistic frameworks, significant gaps persist in understanding m^6^A’s contributions to BPH and prostatitis. Future progress demands technological advancements in m^6^A mapping, innovative model systems, and coordinated clinical translation efforts. By addressing these challenges, we can unlock the full diagnostic and therapeutic potential of m^6^A modulation in prostate-related diseases.

## Glossary

m^6^A: N6-methyladenosine
BPH: benign prostatic hyperplasia
PCa: prostate cancer
ncRNA: non-coding RNA
lncRNA: long non-coding RNA
circRNA: circular RNA
AR: androgen receptor
PSA: prostate-specific antigen
EMT: epithelial-mesenchymal transition
ALKBH5: AlkB homolog 5
SAM: S-adenosylmethionine
METTL3/14: methyltransferase-like 3/14
MTC: methyltransferase complex
WTAP: Wilms tumor 1-associated protein
VIRMA: vir-like m⁶A methyltransferase associated
ZC3H13: zinc finger CCCH-type containing 13
FTO: fat mass and obesity-associated protein
YTHDF1/2/3: YTH domain family 1/2/3
IGF2BP: insulin-like growth factor 2 mRNA-binding protein
hnRNP: heterogeneous nuclear ribonucleoprotein
FMRP: Fragile X Messenger Ribonucleoprotein
PRRC2A: proline rich coiled-coil 2A
TNF-α: tumor necrosis factor-alpha
TGF-β: transforming growth factor-beta
Tregs: regulatory T cells
USF1: upstream stimulatory factor 1
THBS1: thrombospondin 1
G3BP1: GTPase-activating protein binding protein 1
ARPI: androgen receptor pathway inhibition
EGCG: epigallocatechin gallate;NCT, national clinical trial
NF-κB: nuclear factor kappa-light-chain-enhancer of activated B cells
IL-6: interleukin-6
IL-8: interleukin-8
IL-1β: interleukin-1 beta
PIA: proliferative inflammatory atrophy
HGPIN: high-grade prostatic intraepithelial neoplasia
MYC: MYC proto-oncogene
VCAN: versican
PARP: poly (ADP-ribose) polymerase
PD-1: programmed cell death protein 1
PD-L1: programmed death-ligand 1
PTEN: phosphatase and tensin homolog
PI3K: phosphoinositide 3-kinase
AKT: protein kinase B
CRPC: castration-resistant prostate cancer
